# Recent tPA administration can cause pseudo‐hyperargininemia and may mimic arginase deficiency or arginine supplementation

**DOI:** 10.1002/jmd2.12328

**Published:** 2022-10-13

**Authors:** Kristina P. Cusmano‐Ozog, Alicia K. Renck, Christina G. Tise

**Affiliations:** ^1^ Department of Pathology Stanford University Stanford California USA; ^2^ Division of Medical Genetics Stanford University Stanford California USA

**Keywords:** altepase, central indwelling catheter, hyperargininemia, plasma amino acids, propionic acidemia, pseudo‐hyperargininemia, tPA

## Abstract

Individuals suspected of or diagnosed with a rare disorder, including inherited metabolic disorders (IMD), often need frequent and/or urgent vascular access for blood draws and treatment, making central indwelling catheters commonly used devices in this patient population. These indwelling catheters are prone to thrombosis, limiting vascular access. This complication is frequently resolved with the use of altepase, a recombinant tissue plasminogen activator (tPA). This report describes two individuals, one with a known IMD and one undergoing evaluation for an IMD, who were found to have hyperargininemia (>500 μM; reference 10–140 μM) by plasma amino acid (PAA) analysis of a specimen collected ~1.5–3 h after clearance of an indwelling catheter with tPA. In both cases, hyperargininemia resolved with repeat testing, suggesting pseudo‐hyperargininemia secondary to tPA administration. Quantitative amino acid analysis of the administered tPA demonstrated an arginine level of ~200 mM, supporting tPA as the cause of pseudo‐hyperargininemia. Certain formulations of tPA contain high concentrations of arginine, which if not cleared properly can result in marked elevations of arginine, mimicking arginase deficiency or suggesting arginine supplementation. Thus, the possibility of pseudohyperargininemia due to tPA administration should be considered when obtaining PAAs from an indwelling catheter in any individual being evaluated or managed for an IMD.

**Synopsis**

We report pseudo‐hyperargininemia due to tPA administration, mimicking arginase deficiency in two children being evaluated for an IMD.

## 1 INTRODUCTION

Central indwelling catheters, including percutaneous intravenous (IV) central catheter (PICC) lines and port‐a‐cath (port) systems, are not uncommon in individuals with, or undergoing evaluation for, an inherited metabolic disorder (IMD) given the need for frequent and/or urgent vascular access for blood draws and treatment during episodes of metabolic crises. Indwelling catheters are prone to developing clots that can limit vascular access, a complication that is often resolved through the use of altepase, a recombinant tissue plasminogen activator (tPA) that works to break apart the problematic clot. This report describes two individuals, one with known propionic acidemia and one undergoing evaluation for an IMD, who were found to have marked hyperargininemia (>500 μM; reference 10–140 μM) by plasma amino acid (PAA) analysis of a specimen collected ~1.5–3 h after clearance of an indwelling catheter with tPA. In both cases, the hyperargininemia resolved via analysis of subsequent specimens. Quantitative amino acid analysis of the administered tPA demonstrated an arginine level of ~200 mM, supporting tPA as the cause of these incidences of pseudo‐hyperargininemia. Certain formulations of tPA (e.g., Cath‐Flo® Activase®) contain high concentrations of arginine, which if not cleared from a catheter prior to drawing plasma for amino acid analysis, can result in marked elevations of arginine, mimicking arginase deficiency or suggesting arginine supplementation. This iatrogenic cause of elevated arginine has not been previously studied or reported in the literature and should be considered when obtaining PAA analysis from an indwelling catheter in any individual being evaluated or managed for an IMD.

## 2 CASE 1

A 10‐year‐old girl with molecularly confirmed propionic acidemia in need of routine biochemical monitoring laboratory studies presented for a planned hospital admission for port maintenance and consultation with the inpatient Vascular Access Team due to inability to draw from her port. At time 0 h, 5 ml of heparin (100 units/ml) was administered into her single lumen catheter. Due to presumed evidence of a clot within the catheter, 2 mg of intracatheter tPA (specifically Cath‐Flo® Activase® confirmed by inpatient pharmacy) was administered ~1.25 h later, followed by a second dose of tPA administered 3.75 h after the initial dose, with improvement in vascular access. The port was subsequently flushed with 5 ml of heparin (100 units/ml) ~3 h after the second dose of tPA, and PAA were drawn 2 min after the heparin flush. The medical administration record (MAR) within the electronic medical record (EMR) notes that 3 ml normal saline flushes were ordered as needed but not administered.

PAA analysis by liquid chromatography–tandem mass spectrometry (LC–MS/MS)^1^ demonstrated hyperglycinemia (503 μM); however, a marked elevation of arginine (1186 μM; reference 10–140 μM), and an abnormal arginine: ornithine ratio of 23.7 were noted (Table 1). This method of PAA analysis^1^ also incorporates qualitative detection of single reaction monitoring pairs of several other clinically relevant metabolites, including propionylcarnitine (C3), which was elevated and consistent with her diagnosis of propionic acidemia. Free and total carnitine analysis performed on the same specimen demonstrated an acyl: free carnitine ratio of 11.3, also consistent with her known diagnosis of propionic acidemia and provided evidence against a sample mix‐up. Review of the EMR confirmed that no oral or IV arginine was administered during the admission. A call to the Biochemical Genetics Service and family was made to inquire about initiation of new medical formula, medications, or supplements since her last PAA analysis 3 months prior (Table 1); all were denied by both parties. However, both the providers and family reported the use of multiple tPA doses during her recent admission, occurring prior to specimen collection, consistent with the EMR. The remaining tPA used during her admission was obtained from the inpatient pharmacy for PAA analysis, demonstrating an arginine concentration of ~200 000 μM (analytical measurement range: 0–1000 μM; analysis performed on 1:200, 1:400, and 1:800 dilutions). PAA analysis on a specimen collected 40 days after her tPA administration revealed no evidence of hyperargininemia (Table 1).

**TABLE 1:** PAA analysis results of an individual with propionic acidemia before, during, and after an admission for central line maintenance and administration of tPA compared to amino acid analysis of (a) Cath‐Flo® Activase®, (b) plasma from an individual with MELAS on IV arginine therapy, and (c) plasma from an individual with arginase deficiency

	Case 1			Case 2		Cath‐Flo® Activase®	Individuals with MELAS on IV arginine		Individual with arginase deficiency
	3 months prior to admission	Day of tPA administration during admission	40 days after tPA administration	Day of tPA administration during admission	7 days after tPA administration				
Alanine (152–547 μM)	689 (H)	558 (H)	862 (H)	111 (L)	329	ND	461	404	295
Arginine (10–140 μM)	65	1186 (H)	56	576 (H)	79	184 942	1579 (H)	2648 (H)	638 (H)
Citrulline (1–46 μM)	38	27	31	17	35	ND	11 (L)	19	28
Glutamine (254–823 μM)	843 (H)	527	797	333	550	ND	430	385	573
Glycine (127–341 μM)	825 (H)	503 (H)	651 (H)	149	313	ND	196	119 (L)	112 (L)
Isoleucine (22–107 μM)	76	108 (H)	67	28	49	ND	85	59	39
Leucine (49–216 μM)	133	221	123	63	90	ND	128	114	85
Methionine (7–47 μM)	20	30	21	22	26	ND	34	14	60 (H)
Ornithine (10–163 μM)	62	50	41	63	77	ND	174	102	27
Phenylalanine (26–91 μM)	46	57	46	26	41	ND	50	78	44
Tyrosine (24–115 μM)	53	79	55	43	53	ND	35	47	55
Valine (74–321 μM)	130	226	113	118	170	ND	225	198	170
Qualitative priopionylcarnitine (C3) detection (absent)	Present	Present	Present	Absent	Absent	Absent	Absent	Absent	Absent
Arginine:Ornithine	1.0	24	1.4	9.1	1.0	ND	9.1	26	24

Abbreviations: IV, intravenous; MELAS, mitochondrial encephalomyopathy, lactic acidosis, and stroke‐like episodes; ND, not determined; tPA, tissue plasminogen activator.

## 3 CASE 2

A 6 ‐year‐old boy presented to the emergency department with worsening muscle pain and report of dark‐colored urine. Serum creatine kinase (CK) was found to be above the upper limit of detection (>65 000 U/L; reference 39–308 U/L) with a CK‐MB of 2344 ng/ml (reference 0.0–3.6 ng/ml), prompting his admission to the hospital. During his admission, an upper extremity PICC line was placed due to a need for consistent vascular access, and multiple specialists were consulted. Medical Genetics recommended biochemical laboratory studies, including PAA analysis, as well as trio genome sequencing.

Days later, due to presumed evidence of a clot within the catheter, 2 mg of intracatheter tPA (specifically Cath‐Flo® Activase®) was administered at time 0 h with PAA drawn from the PICC line ~90 min afterwards. There is no documented administration of normal saline flushes on that day in the MAR of the EMR. PAA analysis by LC–MS/MS^1^ demonstrated a marked elevation of arginine (576 μM; reference 10–140 μM) with an abnormal arginine: ornithine ratio of 9.1, prompting the Biochemical Genetics Laboratory to obtain additional clinical information due to concern for arginase deficiency. Repeat PAA analysis on a specimen collected 7 days after tPA administration revealed no evidence of hyperargininemia (Table 1). Furthermore, trio genome sequencing did not identify any variants in *ARG1*; however, bialleic variants in a gene associated with an autosomal recessive exercise intolerance and rhabdomyolysis were reported, and he is being managed accordingly.

## 4 DISCUSSION

From this experience, we have come to recognize that some formulations of tPA contain high concentrations of arginine, which if not cleared from a catheter prior to drawing plasma for amino acid analysis, can result in marked elevations of arginine that do not reflect the patient's actual arginine level (i.e., pseudo‐hyperargininemia). The specific brand used in both of these cases was Cath‐Flo® Activase® which contains 77 mg of arginine per 2 mg vial according to the ingredient label.^2^ The reasoning behind manufacturers including arginine as an ingredient in tPA is reported to be for solubility purposes^3^; however, there are other amino acids that could be used to improve the solubility of tPA^4^ suggesting an additional rationale behind the choice of arginine specifically. The choice of arginine for solubilizing tPA likely involves the known role of arginine as a precursor of nitric oxide and subsequent vasodilator. IV arginine is often provided to individuals with mitochondrial encephalomyopathy, lactic acidosis, and stroke like episodes (MELAS) when presenting with symptoms consistent with a stroke‐like episode.^5^ While arginine's mechanism of action would have no effect on the lumen of a catheter lacking an endothelial lining, the arginine component of tPA theoretically could have added value when tPA is administered for the destruction of clots in native blood vessels such as the cerebral vasculature; however the clinical benefit of arginine in tPA solutions is uncertain.^3^, ^6^

It is important that providers be made aware of this iatrogenic cause of elevated argininewhen ordering PAA analysis to be drawn from an indwelling catheter in an individual being evaluated or managed for an IMD, such as a newborn or child with otherwise limited vascular access. Unless recognized as pseudo‐hyperargininemia, this finding may cause unnecessary concern for arginase deficiency and/or concern for unrecommended administration of oral or IV arginine. Conversely, for patients with an IMD for which arginine is commonly supplemented (e.g. MELAS, argininosuccinate lyase deficiency, argininosuccinate synthetase deficiency, ornithine transcarbamylase deficiency) or for patients undergoing a growth hormone‐releasing hormone (GHRH)‐arginine stimulation test to evaluate for growth hormone deficiency, this scenario could result in a provider incorrectly presuming appropriate administration and/or supplement compliance.

The arginine levels in individuals with arginase deficiency are typically 3‐4‐fold the upper limit of normal but can be as high as 10‐fold. ^7^, ^8^ The arginine level in case 1 (1186 μM) is more consistent with arginine levels seen in MELAS patients on IV arginine supplementation (Table 1). With that knowledge, in addition to her known diagnosis of propionic acidemia and prior normal plasma arginine levels, the suspicion for an IV source of arginine far outweighed the concern for a new diagnosis of arginase deficiency. While the arginine level for case 2 (576  μM) was similar to levels observed in individuals with arginase deficiency, the clinical presentation of rhabdomyolysis and lack of molecular variants in *ARG1* was not consistent with arginase deficiency. Recent experience with pseudo‐hyperargininemia from tPA administration through evaluation of case 1 facilitated pertinent inquiry about indwelling catheters and tPA administration upon the laboratory calling the clinical team to obtain additional history regardingthe individual in case 2. However, had these individuals had PAA analysis performed at a laboratory unfamiliar with this association, or had they been a child with a central line without a known diagnosis, the concern for arginase deficiency would have been much higher.

In evaluating patients who screen positive by newborn screening for hyperargininemia, an elevated plasma arginine level in addition to arginine:ornithine ratio ≥1.4 has been suggested as a helpful secondary diagnostic marker in differentiating newborns with arginase deficiency from unaffected newborns.^9^ For this reason, the arginine:ornithine ratio in patients’ samples was calculated to evaluate if this ratio could aid in differentiating individuals with arginase deficiency from those with pseudo‐hyperargininemia secondary to tPA administration and/or MELAS receiving IV arginine therapy (Table 1). Interestingly, the arginine:ornithine ratio was elevated in the specimens from the individual with arginase deficiency, the individuals with MELAS receiving IV arginine therapy, and the individuals with pseudo‐hyperargininemia secondary to tPA administration. This data suggests an elevated arginine:ornithine ratio is not specific for arginase deficiency, as it may represent exogenous administration of arginine (e.g., IV arginineor tPA), and thus should not be used as a differentiating marker in the absence of clinicalin formation.

While having biochemical laboratory studies drawn from a central line is convenient for patients and providers, direct venipuncture and/or sufficient flushing of the central line after tPA administration is required in order to obtain accurate PAA results. It is recommended that 30‐120 minutes following the administration of Cath‐Flo® Activase® and catheter function is restored, that 4–5 ml of blood for those weighing >10 kg (3 ml of blood for those weighing <10 kg) is aspirated to remove residual tPA and that the catheter is flushed with 0.9% NaCl;^10^ however, the exact amount of time and/or flush volume required to clear out any remaining tPA within the lumen of a catheter is unclear and likely varies depending on the clinical scenario. In these two cases, arginine levels of 1168 μM and 576 μM represented a plasma specimen containing approximately 0.05% and 0.03% tPA in case 1 and 2, respectively. If additional time, flushing, and/or ability to perform direct venipuncture is not a viable option after tPA administration to a central line, providers should be aware of the possibility of a falsely elevated arginine level when ordering and interpreting PAA analysis results.

## CONFLICTS OF INTEREST

The authors declare that they have no conflict of interest.

## Data Availability

Data sharing not applicable to this article as no datasets were generated or analyzed during the current study
